# Carbon Starvation Induces the Expression of PprB-Regulated Genes in Pseudomonas aeruginosa

**DOI:** 10.1128/AEM.01705-19

**Published:** 2019-10-30

**Authors:** Congcong Wang, Wenhui Chen, Aiguo Xia, Rongrong Zhang, Yajia Huang, Shuai Yang, Lei Ni, Fan Jin

**Affiliations:** a Department of Chemical Physics, University of Science and Technology of China, Hefei, Anhui, People’s Republic of China; b Hefei National Laboratory for Physical Sciences at the Microscale, University of Science and Technology of China, Hefei, Anhui, People’s Republic of China; c CAS Key Laboratory of Quantitative Engineering Biology, Shenzhen Institute of Synthetic Biology, Shenzhen Institutes of Advanced Technology, Chinese Academy of Sciences, Shenzhen, People’s Republic of China; University of Tartu

**Keywords:** PprB, *Pseudomonas aeruginosa*, biofilm formation, carbon starvation stress

## Abstract

Pseudomonas aeruginosa can cause severe infections in humans. This bacterium often adopts a biofilm lifestyle that is hard to treat. In several previous studies, the PprA-PprB two-component system (TCS), which controls the expression of type IVb pili, BapA adhesin, and CupE fimbriae, was shown to be involved in biofilm formation (M. Romero, H. Silistre, L. Lovelock, V. J. Wright, K.-G. Chan, et al., Nucleic Acids Res 46:6823–6840, 2018, https://doi.org/10.1093/nar/gky324; S. de Bentzmann, C. Giraud, C. S. Bernard, V. Calderon, F. Ewald F, et al., PLoS Pathog 8:e1003052, 2012, https://doi.org/10.1371/journal.ppat.1003052). However, signals or environmental conditions that can trigger the PprA-PprB TCS are still unknown, and the molecular mechanisms of PprB-mediated biofilm formation are poorly characterized. Here, we report that carbon starvation stress (CSS) can induce the expression of *pprB* and genes in the PprB regulon. CSS-induced *pprB* transcription is mediated by the stress response sigma factor RpoS rather than the two-component sensor PprA. We also observed a strong negative regulation of PprB on the transcription of itself. Further experiments showed that PprB overexpression greatly enhanced cell-cell adhesion (CCA) and cell-surface adhesion (CSA) in P. aeruginosa. Specifically, under the background of PprB overexpression, both the BapA adhesin and CupE fimbriae displayed positive effects on CCA and CSA, while the type IVb pili showed an unexpected negative effect on CCA and no effect on CSA. In addition, expression of the PprB regulon genes were significantly increased in 3-day colony biofilms, indicating a possible carbon limitation state. The CSS-RpoS-PprB-Bap/Flp/CupE pathway identified in this study provides a new perspective on the process of biofilm formation in carbon-limited environments.

**IMPORTANCE** Typically, the determination of the external signals that can trigger a regulatory system is crucial to understand the regulatory logic and inward function of that system. The PprA-PprB two-component system was reported to be involved in biofilm formation in Pseudomonas aeruginosa, but the signals triggering this system are unknown. In this study, we found that carbon starvation stress (CSS) induces transcription of *pprB* and genes in the PprB regulon through an RpoS-dependent pathway. Increased PprB expression leads to enhanced cell-cell adhesion (CCA) and cell-surface adhesion (CSA) in P. aeruginosa. Both CCA and CSA are largely dependent on the Bap secretion system and are moderately dependent on the CupE fimbriae. Our findings suggest that PprB reinforces the structure of biofilms under carbon-limited conditions, and the Bap secretion system and CupE fimbriae are two potential targets for biofilm treatment.

## Abstract (precis)

Typically, the determination of the external signals that can trigger a regulatory system is crucial to understand the regulatory logic and inward function of that system. The PprA-PprB two-component system was reported to be involved in biofilm formation in Pseudomonas aeruginosa, but the signals triggering this system are unknown. In this study, we found that carbon starvation stress (CSS) induces transcription of *pprB* and genes in the PprB regulon through an RpoS-dependent pathway. Increased PprB expression leads to enhanced cell-cell adhesion (CCA) and cell-surface adhesion (CSA) in P. aeruginosa. Both CCA and CSA are largely dependent on the Bap secretion system and are moderately dependent on the CupE fimbriae. Our findings suggest that PprB reinforces the structure of biofilms under carbon-limited conditions, and the Bap secretion system and CupE fimbriae are two potential targets for biofilm treatment.

## INTRODUCTION

Pseudomonas aeruginosa is a ubiquitous opportunistic pathogen responsible for many human infections, especially in cystic fibrosis patients (1–3). In many cases of chronic infections, bacteria live in biofilm communities, and increasingly, they are becoming resistant to the human immune system and antibiotic treatments (4–11). Cells in biofilms are typically embedded within a self-produced matrix of extracellular polymeric substances (EPSs) containing polysaccharides, proteins, lipids, and nucleic acids (12–16). Because of its key role in protecting the interior of the community from being killed by antibiotics or immune cells, the dense extracellular matrix has attracted substantial attentions. Numerous studies have pointed out the importance of two extracellular polysaccharides, Pel and Psl, in maintaining functional biofilm structures in P. aeruginosa (17–21). Yet in one previous study, a hyperbiofilm phenotype that was independent of Pel and Psl was identified (22). Cells in this biofilm exhibited overexpression of PprB, decreased type III secretion, and increased drug susceptibility (22). PprB is a two-component response regulator that controls the transcription of numerous genes in P. aeruginosa (22, 23). Moreover, the *pprB* mutant strain was recently shown to form a significantly reduced biofilm in microfluidics systems (24). These results suggest that PprB and its downstream regulated proteins can dominate the formation of biofilm via a Pel- and Psl-independent pathway.

The PprB regulon contains multiple open reading frames, including genes encoding type I secretion system components (*bapA-bapD*), CupE CU fimbriae, and type IVb pili, all of which are positively and directly regulated by PprB at the transcriptional level (22). The *bapA*, *bapB*, *bapC*, and *bapD* (*PA1874-1877*) genes consist of an operon in which *bapA* encodes a large externalized repeat-rich adhesin (22). In addition, BapA protein was found mainly in the supernatant of bacterial culture and associated loosely with the cell surface (22). This raises the question whether BapA can enhance cell adhesion to surfaces. Meanwhile, the CupE fimbriae are cell-surface-associated structures that play an important role in both microcolony and three-dimensional (3D) mushroom formations during biofilm development (25). The type IVb pili are referred to as the tight adherence (Tad) pili and are important in bronchial epithelial cell adhesion and host colonization (26, 27). In P. aeruginosa, the Flp pilin consists of the main structure of the type IVb pilus filament and the *tad* locus proteins (RcpC-TadG) are responsible for ordered secretion, folding, and the assembly of tens of thousands of pilin subunits (28). A previous study had revealed that BapA adhesin, CupE fimbriae, and type IVb pili together contribute to the aforementioned hyperbiofilm phenotype (22).

The phenotypes of PprB overexpression in P. aeruginosa have been well documented. However, in the wild-type strain, the exact external signals or environmental conditions that trigger the PprB pathway remain unknown. Transcriptional studies of *flp*, *rcpC*, and *cupE* promoters under shaking conditions have indicated that these genes are commonly induced in stationary phase (25, 26). In this study, we demonstrated that carbon starvation stress (CSS) triggers the expression of multiple PprB-regulated genes in P. aeruginosa. The induction of PprB-regulated genes is dependent on the RpoS-controlled overexpression of PprB rather than on the signal transduction of the putative sensor kinase PprA. We further demonstrate the roles of type IVb pili, CupE fimbriae, and BapA adhesin in cell-cell adhesion (CCA) and cell-surface adhesion (CSA) by P. aeruginosa. We also observed significant transcriptional increases in PprB regulon genes in colony biofilms after 3 days of cultivation. The CSS-RpoS-PprB-BapA/Flp/CupE signaling pathway determined in this study provides a new perspective on the process of biofilm formation and may be helpful in directing biofilm treatment.

## RESULTS

### *flp* transcription is induced under CSS.

**FIG 1:**
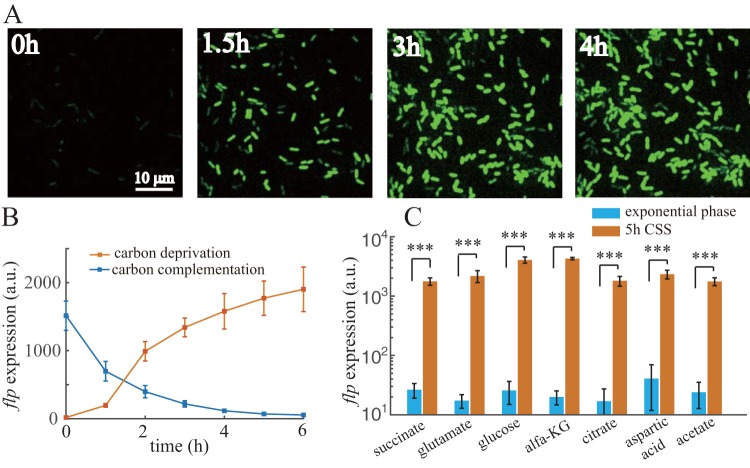
*flp* transcription is induced under CSS. (A) SfGFP time-lapse imaging of *flp* transcriptional reporter cells after carbon deprivation. (B) Resulting expression values of *flp* transcriptional reporter over time after carbon deprivation or *flp* expression over time after carbon complementation of 4-h CSS pretreated cells. (C) Expression values of *flp* transcriptional reporter using different carbon sources at exponential phase or after 5-h carbon deprivation. Statistical analysis used pairwise strain comparisons (*t* test). ***, *P* < 0.001.

Using the superfolder green fluorescent protein (SfGFP), a reporter expression system (see Materials and Methods and Fig. S1 in the supplemental material) was established to assess the transcriptional activity of the *flp* promoter. The reporter strain was first cultured to exponential phase using sodium succinate as the sole carbon source. When cells were washed and introduced to the same medium without sodium succinate, *flp* transcription responded quickly to carbon deprivation. There was a large amount of heterogeneity in SfGFP expressed by the *flp* promoter among cells (Fig. 1A; see also Fig. S2A); the coefficient of variation (CV) for SfGFP expression was 4-fold higher than that of cyan-excitable orange fluorescent protein (CyOFP) expressed by a constitutive promoter (Fig. S2B). SfGFP fluorescence showed an approximately 50-fold (*P* < 0.001) induction after 5 h of carbon deprivation (Fig. 1B). To test whether carbon limitation is the only inducer of *flp* transcription, a bacterial culture that had already experienced 4 h of carbon deprivation was supplemented with 30 mM sodium succinate. SfGFP fluorescence decreased quickly upon succinate addition, and the half-life (1 h) of this decay was approximately the doubling time of cells, indicating that *flp* promoter activity had halted immediately (Fig. 1B). Furthermore, *flp* transcription was similarly induced when we replaced sodium succinate with other types of carbon sources and repeated the carbon deprivation experiment (Fig. 1C). All these results indicate that CSS can induce *flp* transcription in P. aeruginosa.

### PprB is essential for the CSS response of *flp* transcription.

**FIG 2:**
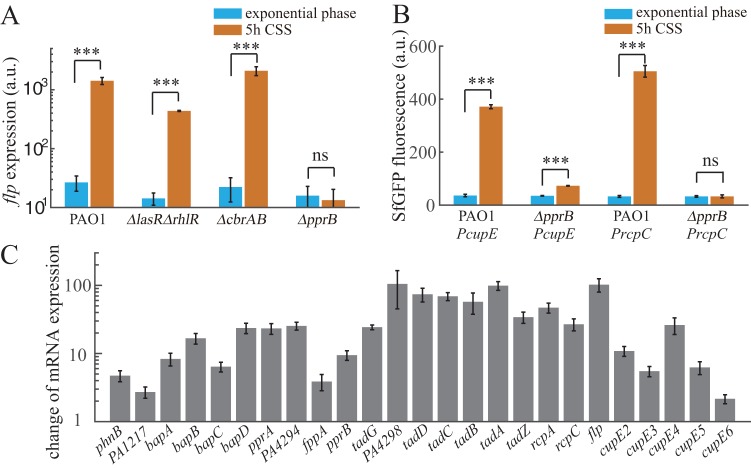
PprB-regulated genes are induced under CSS. (A) Expression values of *flp* transcriptional reporter in different mutants of P. aeruginosa at exponential phase or after 5-h carbon deprivation. (B) Expression values of *cupE* or *rcpC* transcriptional reporters in wild-type or *pprB* mutant strains at exponential phase or after 5-h carbon deprivation. (C) RNA-seq fold change values of mRNA levels of PprB-regulated genes in response to CSS. All data are from three independent experiments and shown as the means ± standard deviations (SDs). Statistical analysis was based on pairwise strain comparisons (*t* test). ***, *P* < 0.001; ns, not significant.

We next investigated the potential regulators involved in controlling *flp* expression under CSS. Previously, *flp* transcription was reported to be mainly dependent on the PprA-PprB two-component regulatory system (26). Moreover, the carbon catabolite control system CbrAB-Crc-CrcZ in P. aeruginosa was found to be involved in the hierarchical management of carbon sources through the regulation of gene expression at both the transcriptional and translational levels (29, 30). In addition, a LasR binding site had been predicted to be upstream of the *flp* coding sequence, suggesting that the quorum sensing system may also be involved. We thus monitored the expression of the *flp* reporter in *pprB*, *cbrA cbrB*, and *lasR rhlR* mutant strains before and after carbon deprivation. *flp* expression in response to CSS was completely eliminated in the *pprB* mutant, while the responses in *cbrAB* and *lasR rhlR* mutants upon CSS were similar to that of the wild-type strain (Fig. 2A). Thus, we concluded that PprB is essential for the CSS-induced expression of *flp*.

### Transcription of *cupE* and *tad* locus is also induced under CSS and is PprB-dependent.

Expression of two gene clusters, the *tad* locus encoding proteins required for type IVb pili assembly and the *cupE* locus encoding nonarchetypal fimbrial subunits, are both controlled by PprB through direct transcriptional regulation (25, 26). We speculated that the expression of these two loci is also induced under CSS. The fluorescence intensities of transcriptional reporters for *cupE* and *rcpC* were monitored in both wild-type and *pprB* mutant strains. Consistent with our speculation, in the wild type, *cupE* and *rcpC* showed 9-fold (*P* < 0.001) and 14-fold (*P* < 0.001) increases, respectively, in expression after carbon deprivation, whereas in the *pprB* mutant, *rcpC* expression in response to CSS was eliminated, while *cupE* expression was only 2-fold (*P* < 0.001) induced (Fig. 2B).

The PprB regulon includes genes involved in *Pseudomonas* quinolone signal (PQS) systems, type 1 secretion systems containing *bapA*, *bapB*, *bapC*, and *bapD*, and the aforementioned type IVb pili and CupE fimbrial assembly systems (22). We further checked the responses of other PprB-regulated genes under CSS using transcriptome sequencing (RNA-seq). Most of the genes within the PprB regulon were upregulated after 6 h of carbon deprivation, with a fold change from 2 to nearly 100 (Fig. 2C). Therefore, the PprA-PprB two-component system was determined to be a key node in the P. aeruginosa CSS response.

### Increased expression of PprB under CSS contributes primarily to the transcriptional induction of PprB-regulated genes.

**FIG 3:**
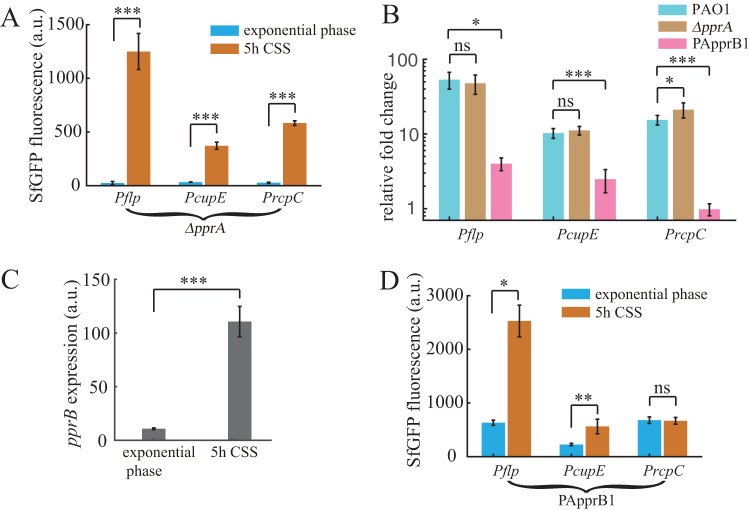
Increased expression of PprB under CSS contributes primarily to the transcriptional induction of PprB-regulated genes. (A) Expression values of *flp*, *cupE*, or *rcpC* transcriptional reporters in the *pprA* mutant strain at exponential phase or after 5-h carbon deprivation. (B) Fold change values of *flp*, *cupE*, or *rcpC* expression upon 5-h carbon deprivation in wild-type, *pprA* mutant, or PApprB1 strains. (C) Expression values of *pprB* transcriptional reporters in the wild-type strain at exponential phase or after 5-h carbon deprivation. (D) Expression values of *flp*, *cupE*, or *rcpC* transcriptional reporters in the PApprB1 (PprB was constitutively overexpressed) strain at exponential phase or after 5-h carbon deprivation. All data are from three independent experiments and shown as the means ± SDs. Statistical analysis was based on pairwise strain comparisons (*t* test). *, *P* < 0.05; **, *P* < 0.01; ***, *P* < 0.001; ns, not significant.

We then focused on how the PprA-PprB system responds to CSS. A previous study indicated that PprA functions as a sensor kinase, responsible for PprB phosphorylation in response to external signals (23). Thus, we considered that the CSS signal should be transmitted through PprA. However, reporters in a *pprA* mutant exhibited similar responses to CSS as in the wild type (Fig. 3A and B). Therefore, although PprB mediates the transcriptional response of related genes, the CSS signal is not transmitted through PprA. Another possibility is that the CSS signal may trigger the expression of *pprB* (which was already observed in the RNA-seq result), thereby upregulating the expression of related genes. We further constructed a *pprB* transcriptional reporter in P. aeruginosa and investigated its response to CSS. Consistent with the RNA-seq data, the fluorescence of the *pprB* reporter in wild-type cells was increased approximately 10-fold (*P* < 0.001) upon 5 h of carbon starvation (Fig. 3C).

To further check whether the transcriptional inductions of PprB-regulated genes under CSS could be explained by increased PprB expression, reporters of *flp*, *cupE*, and *rcpC* were measured in a PApprB1 strain in which *pprB* was constitutively overexpressed. This PApprB1 strain was constructed by introducing the *pprB* gene into the chromosomal attTn*7* site of the *pprB* knockout strain. Notably, the exogenously introduced *pprB* gene was driven by the arabinose-inducible promoter *P_BAD_*. Exponential-phase expressions of the reporters increased more than 10-fold in PApprB1 compared to that in the wild type (Fig. 2B and 3D), consistent with the fact that PprB positively controls transcription of these genes. However, CSS failed to induce the same expression changes of *flp*, *cupE*, and *rcpC* in PApprB1. Only 3-fold (*P* < 0.05), 1.5-fold (*P* < 0.01), and 0-fold increases for *flp*, *cupE*, and *rcpC*, respectively, were observed in PApprB1 (Fig. 3B and D), in contrast to the corresponding 50-fold (*P* < 0.001), 9-fold (*P* < 0.001), and 14-fold (*P* < 0.001) increases in PAO1 (Fig. 3B). Therefore, we conclude that transcriptional induction of *cupE*, *rcpC*, and *flp* under CSS is primarily driven by increased expression of PprB.

### Increased expression of PprB under CSS is controlled by RpoS.

**FIG 4:**
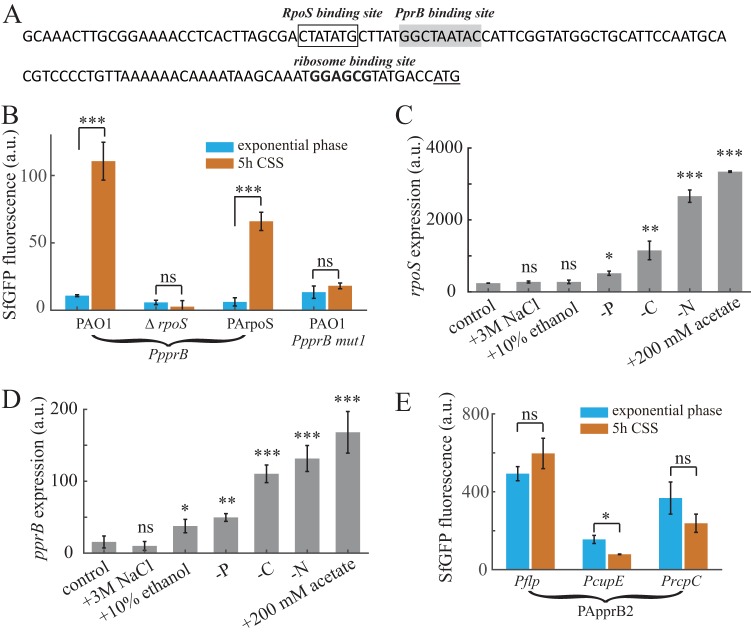
Increased expression of PprB under CSS is controlled by RpoS. (A) Promoter region of the *pprB* gene. Putative RpoS or PprB binding sites are framed or greyed. The ribosome binding site is shown in boldface and the translational start codon is underlined. (B) Expression values of *pprB* or p*pprB*-mut1 (RpoS binding sequence CTATATG was mutated to GGGTATG) transcriptional reporters in the wild-type, *rpoS* mutant, or PArpoS (Δ*rpoS*, *rpoS* complement at genomic attTn*7* site) strains at exponential phase or after 5-h carbon deprivation. Expression values of *pprB* (C) or *rpoS* (D) transcriptional reporter under different stress conditions. (E) Expression values of *flp*, *cupE*, or *rcpC* transcriptional reporters in the PApprB2 (Δ*rpoS*, PprB overexpression) strain at exponential phase or after 5-h carbon deprivation. Data are from three independent experiments and shown as the means ± SDs. Statistical analysis was based on pairwise strain comparisons (*t* test). *, *P* < 0.05; **, *P* < 0.01, ***, *P* < 0.001; ns, not significant.

We continued to search for regulators that control the expression of PprB. In P. aeruginosa, the stress response sigma factor RpoS has been reported to enhance carbon starvation tolerance (31, 32); thus, it is reasonable to speculate that RpoS is involved in the regulation of genes with altered expression during CSS. In the *rpoS* mutant, SfGFP fluorescence from the *pprB* transcriptional reporter was barely detectable under CSS, while complementing the *rpoS* mutation in PArpoS restored SfGFP fluorescence (Fig. 4B), indicating the pivotal role that RpoS plays in *pprB* transcription. Based on the previously identified RpoS-dependent promoter consensus (33), a putative RpoS binding site (CTATATG) was identified in the *pprB* promoter sequence (Fig. 4A). The p*pprB*-mut1 reporter, whose RpoS binding site was mutated (CTATATG to GGGTATG), also failed to respond to CSS in the wild-type strain (Fig. 4B). Moreover, under conditions in which *rpoS* expression was induced (CSS, nitrogen starvation, and acetate stress), *pprB* expression was also correspondingly increased (Fig. 4C and D); the expression of *pprB* and *rpoS* displayed a good positive correlation (correlation coefficient = 0.95). All these results strongly suggest that RpoS directly regulates the transcription of *pprB* and that the accumulation of intracellular RpoS mediates the CSS-induced transcriptional responses of *pprB* and PprB-regulated genes.

Expression of *flp*, *cupE*, and *rcpC* was further examined in strain PApprB2, in which *rpoS* was knocked out and *pprB* was overexpressed by the *P_BAD_* promoter. All three reporters in the PApprB2 strain displayed decreased expression with respect to that in PApprB1, especially under CSS (Fig. 4E). Transcriptional induction of *flp* and *cupE* in PApprB1 by CSS was completely abrogated (Fig. 4E). We also identified several putative RpoS binding sequences within *flp*, *cupE*, and *rcpC* promoters (see Fig. S3). These results suggest that RpoS can also control the transcription of PprB-regulated genes through a PprB-independent way, possibly through direct binding to the *flp*, *cupE*, and *rcpC* promoters.

### PprB overexpression enhances CCA in P. aeruginosa.

We next investigated the possible effects of *pprB* upregulation on the physiology of P. aeruginosa. Overexpression of PprB was reported to result in a hyperbiofilm phenotype that was dependent on type IVb pili, the CupE fimbriae, and the BapA adhesin (PA1874), but the exact mechanism by which this occurs remains unclear (22). During biofilm formation in flow chambers, bacteria are engaged in a dynamic process of growth and detachment, and the rates of bacterial growth and detachment within a biofilm are the two key factors that determine the resultant biomass. As the growth of *pprB* overexpressing cells is not faster than that of wild-type cells (see Fig. S4), we speculated that the hyperbiomass phenotype may have been due to enhanced cell-to-cell or cell-to-surface adhesion, both of which may reduce the detachment rate.

**FIG 5:**
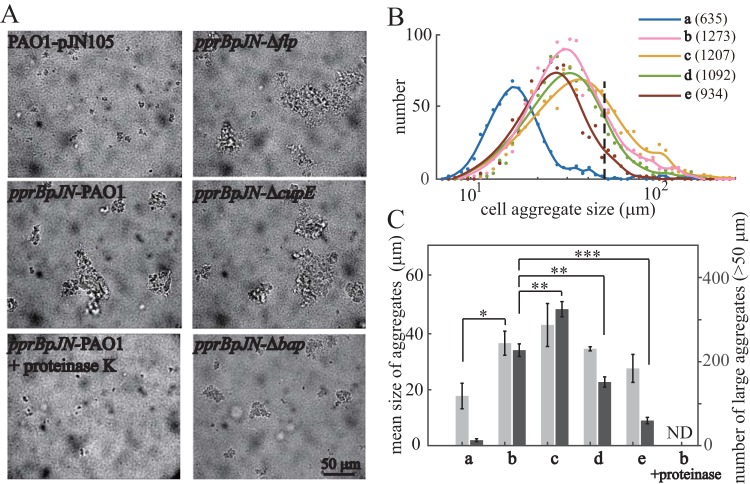
PprB overexpression enhances CCA in P. aeruginosa. (A) Bright-field images of the exponential-phase bacterial cultures. (B) Size distribution of cell aggregates; total numbers of cell aggregates counted for each strain are in parentheses after strain labels. The distribution curves are the smoothing result of original data points using a smoothing spline method. The black dashed line indicates the position where cell aggregate size equals 50 μm in diameter. (C) Mean sizes of cell aggregates (light gray) and numbers of large aggregates (dark gray, cell aggregates >50 μm in diameter). Mean aggregate sizes are from three independent experiments and shown as the means ± SDs. Errors of large aggregates numbers are estimated from Poisson counts by $\sqrt{N}$, where *N* is the number of large aggregates. ND, not detected. Statistical analysis was based on pairwise comparisons between corresponding data in *pprB*pJN-PAO1 and data in other strains (*t* test). *, *P* < 0.05; **, *P* < 0.01; ***, *P* < 0.001. Strains used in panels B and C are as follows: a, PAO1-pJN105; b, *pprB*pJN-PAO1; c, *pprB*pJN-Δ*flp*; d, *pprB*pJN-Δ*cupE*; e, *pprB*pJN-Δ*bap*.

The CCA of bacteria was estimated by observing bacterial aggregate formation in shaking cultures at the exponential phase. Both the mean size and number of bacterial aggregates in the PprB overexpression strain were approximately twice that of the wild-type strain (Fig. 5A and B). Additionally, bacterial aggregates disappeared completely after a 30-min incubation with proteinase K at 37°C (Fig. 5A). Thus, CCA was enhanced by PprB overexpression, and the PprB-regulated proteins may directly contribute to CCA. We then monitored CCA in PprB overexpression strains whose *flp*, *cupE*, or *bap* was deleted. The mean sizes of bacterial aggregates in these mutants displayed small differences from that in the wild type (Fig. 5C, light gray). However, the numbers of large bacterial aggregates (cell aggregates >50 μm in diameter) differed greatly between these mutant strains according to the size distribution curves (Fig. 5B). Under a PprB overexpression background, the number of large bacterial aggregates was (i) increased by 43% (*P* < 0.01) when *flp* was deleted and (ii) reduced by 33% (*P* < 0.01) or 74% (*P* < 0.001) when *cupE* or *bap* was deleted, respectively (Fig. 5C, dark gray). These results demonstrate that the Bap adhesin secretion system and CupE fimbriae partially contribute to CCA, while the type IVb pili have a negative effect on CCA.

Interestingly, cell clustering was only observed when arabinose was added at the very start of the bacterial inoculation. When arabinose was added at the exponential phase (optical density at 600 nm [OD_600_] of ∼0.5), few clusters were seen, and we also did not observe any clusters during the carbon deprivation experiment (in which cells can hardly grow). This phenomenon suggests that there is a currently unknown relationship between bacterial clustering and cell division.

### PprB overexpression enhances CSA in exponentially growing P. aeruginosa.

**FIG 6:**
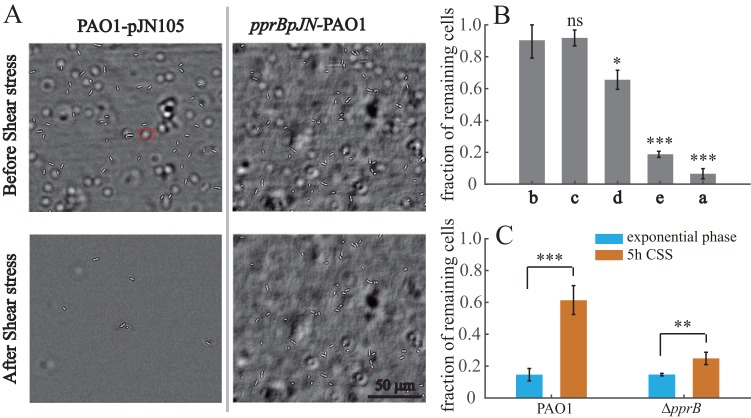
PprB overexpression enhances CSA in P. aeruginosa. (A) Bright-field images of the wild type or cells overexpressing PprB before and after exposure to a 5-min shear stress (70 Pa) in a microfluidic channel. The white halo marked by a red rectangle is a bacterial cell adhered to the upper surface of the microfluidic channel. (B) Fractions of cells remaining adhered to the surface after exposure to a 5-min shear stress (70 Pa). Strains used are as follows: a, PAO1-pJN105; b, *pprB*pJN-PAO1; c, *pprB*pJN-Δ*flp*; d, *pprB*pJN-Δ*cupE*; e, *pprB*pJN-Δ*bap*. (C) Fractions of wild-type or *pprB* mutant cells remaining adhered to the surface after exposure to a 5-min shear stress (70 Pa); cells were from the exponential phase or treated with 5-h CSS. Data in panels B and C are from three independent experiments and shown as the means ± SDs. Statistical analysis was based on pairwise strain comparisons (*t* test). *, *P* < 0.05; **, *P* < 0.01; ***, *P* < 0.001; ns, not significant.

The CSA of bacteria was estimated using a microfluidic device. Bacterial cultures were injected into the device in the absence of flow and incubated for 20 min to enable initial adhesion. Then, we washed the microfluidic channel with a 70 Pa shear stress for 5 min, and the numbers of cells on the surface before and after shear stress were counted. The effect of *pprB* overexpression on CSA was investigated first. Fluidic shear eliminated most of the adhered cells in the wild-type strain. In contrast, cells overexpressing PprB appeared to be largely unaffected, with only a few incompletely adhered cells eliminated (Fig. 6A), while the remaining cells remained adhered to the surface, even at shear stress of 1,000 Pa. In a PprB overexpression background, the fractions of remaining cells after shear stress were (i) not affected when *flp* was deleted or (ii) reduced by 27% (*P* < 0.05) or 79% (*P* < 0.001) when *cupE* or *bap* was deleted, respectively (Fig. 6B). Thus, both the CupE fimbrial and the Bap secretion systems are involved in the enhanced CSA by PprB overexpression.

We further monitored the CSA of the wild-type and *pprB* mutant cells before and after carbon deprivation. As expected, the fraction of remaining cells in the wild-type strain was increased 4-fold (*P* < 0.001) upon CSS, in contrast to the 50% (*P* < 0.01) increase observed in the cells of the *pprB* mutant (Fig. 6C). Taken together, our results confirm that PprB overexpression can enhance bacterial CCA and CSA, which probably leads to the hyperbiofilm phenotype.

Interestingly, although type IVb pili were reported to be essential for the formation of the previously reported hyperbiofilm phenotype (22), this cell surface structure showed no contribution to bacterial CSA (Fig. 6B) and showed a negative effect on bacterial CCA (Fig. 5C). The function of type IVb pili in biofilm formation remains unclear at this time.

### PprB negatively regulates the transcription of itself.

**FIG 7:**
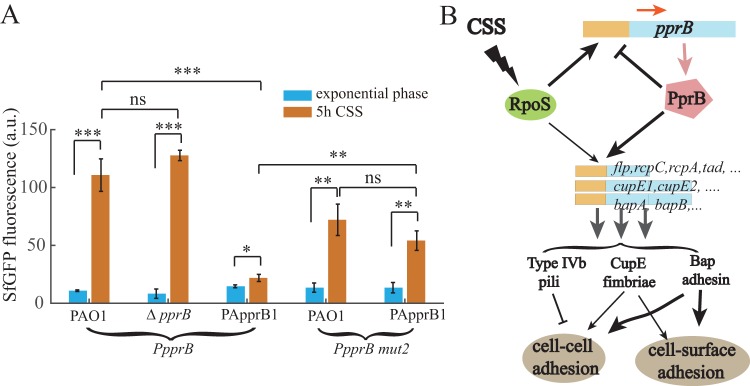
PprB negatively regulates the transcription of itself, and model of CSS responses of PprB-regulated genes through RpoS. (A) Expression values of *pprB* or p*pprB*-mut2 (PprB binding sequence GGCTAATAC was mutated to GGCGGGTAC) transcriptional reporters in the wild type or *pprB* mutant or PApprB1 (PprB was constitutively overexpressed) strains at exponential phase or after 5-h carbon deprivation. Data are from three independent experiments and shown as the means ± SDs. Statistical analysis was based on pairwise strain comparisons (*t* test). *, *P* < 0.05; **, *P* < 0.01; ***, *P* < 0.001. (B) Schematic representation of the RpoS-PprB-Flp/CupE/Bap/Tad system and its signaling cascade in response to CSS. CSS induces the expression of PprB-regulated genes by triggering the expression of PprB. RpoS mediates the CSS signal induction of PprB transcription. Expression of CupE fimbriae (moderately) and Bap adhesin (largely) enhances bacterial CCA and CSA, the type IVb pili have a negative effect on CCA. PprB negatively regulates the transcription of itself.

Many transcriptional regulators in bacteria exhibit self-regulation activities, either positive or negative. PprB was reported to bind to the *pprB* promoter region (26), in which a putative PprB binding site (GGCTAATAC) was mapped on the basis of a previously predicted PprB recognition consensus (Fig. 4A). The PprB binding site stands immediately downstream of an RpoS site, suggesting a negative effect of PprB on *pprB* transcription due to the steric interference with RNA polymerase. To verify this assumption, we measured the fluorescence of the *pprB* reporter during exponential phase or under carbon deprivation conditions in both the *pprB* mutant and overproducing strains. Under CSS, the activity of the *pprB* reporter in the *pprB* mutant strain was similar to that in the wild-type strain, while in PApprB1 cells, it was 20% (*P* < 0.001) of that in the wild-type strain (Fig. 7A). Moreover, the expression of the p*pprB*-mut2 reporter whose PprB binding site was mutated (GGCTAATAC to GGCGGGTAC) was measured in the wild-type and PApprB1 strains. In response to CSS, the p*pprB*-mut2 reporter expression in PApprB1 increased 4-fold (*P* < 0.01), similar to the 5.5-fold increase (*P* < 0.01) found in the wild-type strain (Fig. 7A). All these results confirm that *pprB* transcription is under direct negative control of PprB. The model of CSS responses of PprB-regulated genes through RpoS is presented in Fig. 7B.

### Expression of PprB-regulated genes is upregulated in colony biofilms.

**FIG 8:**
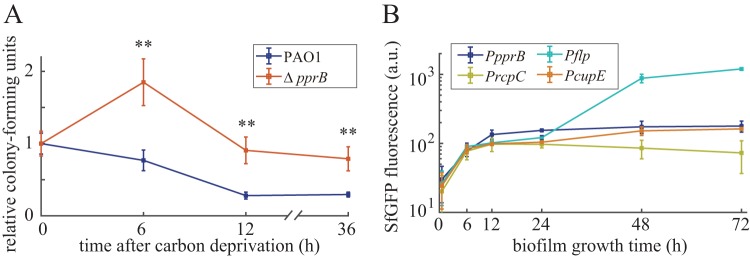
(A) Relative CFU counts of wild-type and *pprB* mutant cells after carbon deprivation for 0, 6, 12, and 36 h under shaking conditions at 37°C. CFU data of each strain were normalized by data at 0 h. Data are from three independent experiments and shown as the means ± SDs. Statistical analysis was based on pairwise comparisons between PAO1 and the *pprB* mutant (*t* test). **, *P* < 0.01. (B) Time-dependent expression curves of *pprB*, *flp*, *rcpC*, or *cupE* genes in the wild-type cells grown in colony biofilms at 37°C.

According to evolutionary theory, the induction of genes under a specific condition should be beneficial for the bacteria, whether through improved fitness or from enhanced competitive advantage over other organisms. We monitored the CFU of the wild-type and *pprB* mutant strains under CSS in shaking cultures. Contrary to our expectation, CFUs of the *pprB* mutant were larger than those of the wild type after CSS treatment (Fig. 8A). Despite the fact that bacteria cannot increase their biomass without carbon supplementation, we found that cells still divided into smaller daughter cells under CSS (see Fig. S5), which may contribute to the increased CFU of the *pprB* mutant after 6-h carbon deprivation. The decreased cell size of P. aeruginosa under CSS is consistent with previously reported cell size reduction of bacteria entering into stationary phase (34, 35), suggesting that cells under CSS are in a stationary-phase-like growth state. Since biofilm is considered the natural form of existence of P. aeruginosa, and according to the previously found biological filtration effect of biofilm (36), cells in the deep inner regions of biofilm may encounter CSS as the biofilm grows and thickens. We detected the expression of both *pprB* and PprB-regulated genes in P. aeruginosa colony biofilms. All the observed genes displayed thorough induction of expression after a 72-h incubation (Fig. 8B), indicating the probable involvement of PprB in biofilm development. Taking into consideration that PprB downstream proteins are involved in CCA and CSA, this CSS-RpoS-PprB-BapA/Flp/CupE signaling pathway may help reinforce the structure of P. aeruginosa biofilms.

## DISCUSSION

The PprA-PprB two-component system has been studied for more than 10 years, and the PprB regulon containing multiple functional gene clusters was characterized several years ago (22, 23, 25, 26). With regard to the physiological role of PprB in bacteria, previous studies have mainly focused on the phenotypes of the *pprB* overexpression strains, which, compared with the wild-type strain, have shown increased cell membrane permeability and aminoglycoside sensitivity, decreased cellular cytotoxicity and virulence in flies, and better biofilm formation (22, 23). However, except for one recent report that *pprB* knockout leads to reduced biofilm in a microfluidic system (24), very few studies have focused on the phenotypes of the *pprB* mutant strain. This is partially due to the fact that the signals and environmental conditions that may trigger the PprA-PprB system remain unclear. Generally, the determination of the external signals that can trigger a regulatory system is crucial to understand the regulatory logic and inward function of that system. In this paper, we provide evidence that the PprB-regulated genes are induced via CSS. In particular, the induction of the transcription of PprB-regulated genes is dependent on the increased expression of PprB rather than on the activation of the PprA kinase. We further demonstrate that the stress response sigma factor RpoS controls the induction of *pprB* transcription.

In many organisms, the small-molecule alarmone (p)ppGpp is the main effector of the stress response that takes place during starvation (37). The (p)ppGpp synthase RelA senses the lack of tRNA aminoacylation during carbon starvation and translates the carbon starvation signal into one for the synthesis of intracellular (p)ppGpp (38). RelA-dependent (p)ppGpp accumulation was also demonstrated in Streptococcus suis under CSS (39). In Escherichia coli, (p)ppGpp positively affects the intracellular level and function of RpoS through the multifaceted regulation of transcription, translation, proteolysis, and activity (40), thereby tying the CSS signal to the response of the RpoS regulon. As most of the genes in the (p)ppGpp-RpoS system of E. coli can also be found in the P. aeruginosa genome, it is possible that the RpoS-dependent *pprB* transcriptional response observed in this study was achieved through the same (p)ppGpp-RelA stress-sensing mechanism. This hypothesis was supported by our subsequent experiments, in which CSS failed to induce the expression of *flp*, *cupE*, *rcpC*, *pprB*, and *rpoS* in a *relA* mutant strain (see Fig. S6 in the supplemental material). In addition, CSS is not the only signal that can induce PprB expression. Nitrogen starvation stress and acetate stress, two other signals that can trigger the RpoS stress-response system, also induce the transcription of *pprB* (Fig. 4C and D). Thus, signals facilitating the accumulation of intracellular RpoS are probably the signals that activate the expression of PprB and PprB-regulated genes.

**FIG 9:**
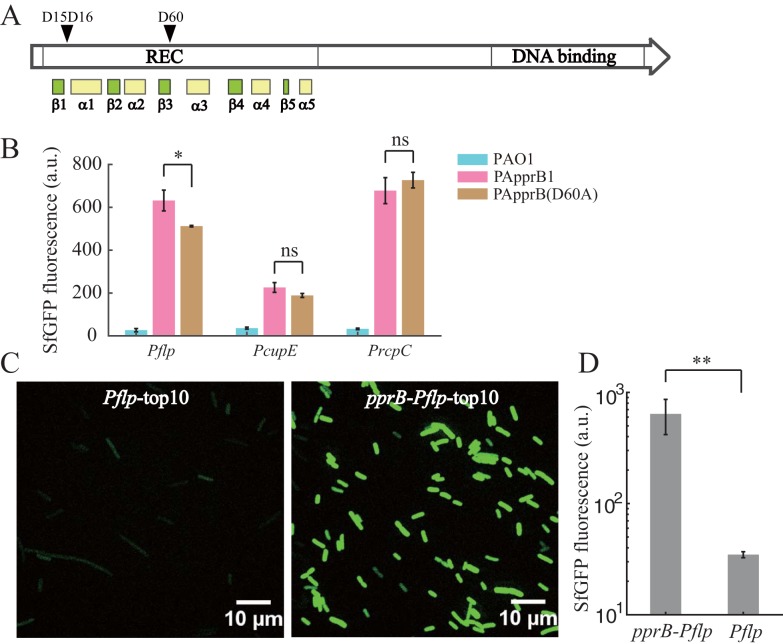
Transcriptional regulatory activity of PprB is phosphorylation independent. (A) Analysis of the phosphorylation site in the REC domain of PprB. D15 and D16 are the two acidic residues within the loop that connects β1 and α1, which are involved in Mg^2+^ ion binding, and D60 is the phosphorylation site at the end of the third β-strand. (B) Expression values of *flp*, *cupE*, and *rcpC* transcriptional reporters in PAO1, PApprB1, or PApprB(D60A) strains. (C) SfGFP images of *flp* transcriptional reporter in E. coli (TOP10 strain) with or without *pprB* overexpression. (D) Expression levels of *flp* transcriptional reporter in E. coli (strain TOP10) with or without *pprB* overexpression. Statistical analysis was based on pairwise strain comparisons (*t* test). *, *P* < 0.05; **, *P* < 0.01; ns, not significant.

PprA was previously reported to be the cognate kinase for PprB (23). However, PprB is still active in the *pprA* mutant strain according to the fact that *pprA* knockout failed to eliminate or reduce the CSS response of PprB-regulated genes. One possibility is that other kinases or small phosphor donor molecules such as acetyl phosphate are responsible for PprB phosphorylation; this situation allows PprB to respond to other kinds of signals in addition to the RpoS-related stress signals. However, we have not found any alternative kinases or phosphor donors that phosphorylate PprB. An alternative explanation is that the regulatory activity of PprB is independent of PprB phosphorylation, which is contrary to our knowledge of two-component systems (41–43). The active site of the common response regulator is composed of an aspartic acid residue at the end of the third β-strand (receives the phosphoryl group from the respective HK) and two acidic residues (usually an aspartate/glutamate and aspartate) within the loop that connects β1 and α1, which are involved in Mg^2+^ ion binding (44). These three signature residues were found at the corresponding sites in the REC domain of PprB (Fig. 9A). We then mutated PprB at the phosphorylation site (D60A) and overexpressed PprB(D60A) in the *pprB* mutant strain to see whether PprB-regulated genes could be upregulated. Contrary to our expectations, the expression of *flp*, *cupE*, and *rcpC* was upregulated greatly by PprB(D60A), and their expression levels were similar to that in the PApprB1 strain (Fig. 9B). We also measured the expression of *flp* transcriptional reporter in E. coli (TOP10 strain) with or without PprB expression. Overexpression of PprB in E. coli led to a 20-fold (*P* < 0.01) increase of *flp* expression (Fig. 9C and D). All of these results strongly suggest that phosphorylation is not necessary for the transcriptional regulatory activity of PprB.

It is worth noting that, in P. aeruginosa, there is another cell surface-associated fimbria named type IVa pilus that is essential for bacterial twitching and swarming and is also important for biofilm formation (45). In addition, surface motilities of P. aeruginosa are greatly affected by using different carbon sources (46, 47), indicating the possible involvement of carbon sources in the regulation of type IVa pili expression. Interestingly, according to our RNA-seq result, the expression of most type IVa pilus-encoding genes is downregulated upon CSS (see Table S1), in sharp contrast to the significant upregulation of the expression of type IVb pilus-encoding genes (Table S1, Fig. 2C). The opposite regulation of type IVa and type IVb pili under CSS may reflect the distinct functions of the two fimbriae in bacterial adaptation to CSS.

## MATERIALS AND METHODS

### Bacterial strains and growth conditions.

The strains and plasmids used in this study are listed in Table 1. Unless otherwise stated, 1 ml of bacterial cultures was cultivated in 5.5-ml polystyrene 12-mm by 75-mm round-bottom tubes (Falcon 352054) with shaking (250 rpm) at 37°C. Cells were grown in FAB minimal medium (48) supplemented with 30 mM sodium succinate (FABS) or other carbon sources (i.e., 30 mM sodium glutamate, 30 mM glucose, 10 mM alpha-ketoglutaric acid [alfa-KG], 30 mM sodium citrate, 30 mM aspartic acid, or 30 mM sodium acetate). To prevent plasmid loss, 30 μg/ml gentamicin was added to media for cultivation of the strains containing transcriptional reporter plasmids or pJN105-derivative vectors. LB medium was used throughout the DNA cloning experiments. The Escherichia coli TOP10 strain was used for standard genetic manipulations.

**TABLE 1:** Strains, plasmids, and primers used in this study

Strain, plasmid, or primer	Description or sequence (5′→3′)	Origin or reference
Strains		
E. coli		
TOP10	F^−^ *mcrA* (*mrr hsdRMS–mcrBC*) 80*lacZ* M15 *lacX74 recA1 araD139* (ara–leu)7697 *galU galK rpsL*(Str^r^) *endA1 nupG*	Invitrogen
P. aeruginosa		
PAO1	Wild-type strain	J. D. Shrout
Δ*pprB*	nonpolar *pprB* deletion in PAO1	This study
Δ*pprA*	nonpolar *pprA* deletion in PAO1	This study
Δ*rpoS*	nonpolar *rpoS* deletion in PAO1	Kangming Duan group
Δ*flp*	nonpolar *flp* deletion in PAO1	This study
Δ*cupE*	nonpolar *cupE* deletion in PAO1	This study
Δ*bapA*	nonpolar *bapA* deletion in PAO1	This study
Δ*relA*	nonpolar *relA* deletion in PAO1	This study
Δ*lasR*Δ*rhlR*	nonpolar *lasR* and *rhlR* deletions in PAO1	J. D. Shrout
Δ*cbrA*Δ*cbrB*	nonpolar *cbrA* and *cbrB* deletions in PAO1	This study
PApprB1	Δ*pprB*, *araC-P_BAD_-pprB*-miniTn*7*	This study
PApprB(D60A)	Δ*pprB*, *araC-P_BAD_-pprB(D60A)*-miniTn*7*	This study
PApprB2	Δ*rpoS*, *araC-P_BAD_-pprB*-miniTn*7*	This study
PArpoS	Δ*rpoS* P*rpoS-rpoS*-miniTn*7*	This study
PAO1 pJN105	PAO1 strain containing pJN105 void vector, Gm^r^	This study
*pprB*pJN-PAO1	PAO1 strain containing *pprB*-pJN105, *pprB* expression under the control of arabinose concentration, Gm^r^	This study
*pprB*pJN-Δ*flp*	Δ*flp* strain containing *pprB*-pJN105, Gm^r^	This study
*pprB*pJN-Δ*cupE*	Δ*cupE* strain containing *pprB*-pJN105, Gm^r^	This study
*pprB*pJN-Δ*bapA*	Δ*bapA* strain containing *pprB*-pJN105, Gm^r^	This study
Plasmids		
pUCPgfps	Cloning vector for transcriptional reporter, RNAseIII-RBS2-*sfgfp*-T0T1-J23102-RBS2-*cyofp*-T-PUCP20, Gm^r^	This study
p*flp*-PUCPgfps	Transcriptional reporter plasmid of *flp*, Gm^r^	This study
p*cupE*-PUCPgfps	Transcriptional reporter plasmid of *cupE1*, Gm^r^	This study
p*rcpC*-PUCPgfps	Transcriptional reporter plasmid of *rcpC*, Gm^r^	This study
p*pprB*-PUCPgfps	Transcriptional reporter plasmid of *pprB*, Gm^r^	This study
p*pprB*-mut1	Transcriptional reporter plasmid of *pprB*, with RpoS binding site mutated from CTATATG to GGGTATG, Gm^r^	This study
p*pprB*-mut2	Transcriptional reporter plasmid of *pprB*, with PprB binding site mutated from GGCTAATAC to GGCGGGTAC, Gm^r^	This study
pex18ap	oriT^+^*sacB*^+^; gene replacement vector with MCS from pUC18; Ap^r^	46
pex18gm	oriT^+^*sacB*^+^; gene replacement vector with MCS from pUC18; Gm^r^	46
pFLP2	*sacB*^+^; Flp recombinase-expressing *bhr* vector; Ap^r^	46
*flp*-gen-pex18ap	In-frame deletion of *flp* cloned into HindIII-XbaI sites of pex18ap; Ap^r^, Gm^r^	This study
*pprB*-gen-pex18ap	In-frame deletion of *pprB* cloned into HindIII-XbaI sites of pex18ap; Ap^r^, Gm^r^	This study
*cupE*-pex18gm	In-frame deletion of *cupE* operon (*cupE1-cupE6*) cloned into pex18gm; Gm^r^	This study
*bapA*-pex18gm	In-frame deletion of *bap* operon (*bapA-bapD*) cloned into pex18gm; Gm^r^	This study
*pprA*-pex18gm	In-frame deletion of *flp* cloned into pex18gm; Gm^r^	This study
*P_BAD_-pprB*-Tn*7*	*araC-P_BAD_-pprB*-miniTn*7*, *pprB* complementary plasmid for chromosomal insertion at attTn*7* site, *pprB* expression is controlled by *P_BAD_* promoter	This study
*P_BAD_-pprB(D60A)*-Tn*7*	*araC-P_BAD_-pprB*(D60A)-miniTn*7*, complementary expression of PprB(D60A) for chromosomal insertion at attTn*7* site, *pprB*(D60A) expression is controlled by *P_BAD_* promoter	This study
*P_rpoS_-rpoS*-Tn*7*	*rpoS* complementary plasmid for chromosomal insertion at attTn*7* site, *rpoS* expression is controlled by its own promoter	This study
*pprB*-pJN105	*pprB* overexpression vector in pJN105, *pprB* expression is controlled by *P_BAD_* promoter	This study
Primers		
miniTn*7* cloning primers		
pprB-OL-Tn7-F	CCTGCAAGGCCTATGCTACTCCGTCAAGCCGT	
pprB-ol-Tn7-R	GAGCTCACTAGTTCAGTGCACCACCGCTCCGC	
pprB-D60A-F	CTGGTCATCTGCGCCCTCTACCTGGGCCAGGACAAC	
pprB-D60A-R	CCCAGGTAGAGGGCGCAGATGACCAGGCCGATGTT	
Tn7-OL-araC-R	GACGGAGTAGCATAGGCCTTGCAGGCCAACCAGA	
Tn7-OL-pprB-F	GGTGGTGCACTGAACTAGTGAGCTCATGCATGATCGAAT	
rpos-ol-Tn7-F	TCTGGTTGGCCTGCAAGGCCTTCCATTGCCTTCCGCTTCGGCTG	
rpos-ol-Tn7-R	CGATCATGCATGAGCTCACTAGTTCACTGGAACAGCGCGTCACT	
Tn7-ol-rops-R	GGAAGGCAATGGAAGGCCTTGCAGGCCAACCAGATAA	
Tn7-ol-rpos-F	CGCTGTTCCAGTGAACTAGTGAGCTCATGCATGATCG	
pJN105 cloning primers		
pJN105-F	GAGCTCCAATTCGCCCTATAGTGAG	
pJN105-rbs-R	GCTTAATCTCCTTCTTTTCCACAGCGAATTCGCTAGCCCAAAAAAACGG	
pprB-OL-PJN-F	GCTGTGGAAAAGAAGGAGATTAAGCATGGACAAACCGGCCTCG	
pprB-OL-PJN-R	CTCACTATAGGGCGAATTGGAGCTCTCAGTGCACCACCGCTCC	
Gene knockout primers		
cbrAB-up-HindIII-F	GAGATAAGCTTACTTCGGTTCCCTGGTGG	
cbrAB-up-BamHI-R	GAGATGGATCCGAGGTAGGTGACGCTGATCA	
cbrAB-dn-BamHI-F	GAGATGGATCCGACTCGTAACACCCTGCAAC	
cbrAB-dn-XbaI-R	GAGATTCTAGAAGGATCTCGACGACCTTGAC	
flp-up-HindIII-F	GAGATAAGCTTGTCCTTTTCCTGGTTCGAGC	
flp-up-BamHI-R	GAGATGGATCCCAGGTTCTTCATTCTTGTTTGCTC	
flp-dn-BamHI-F	GAGATGGATCCGCTCCGACAGCGAACTGAC	
flp-dn-XbaI-R	GAGATTCTAGAGTTGCATCAGTACGCGGATC	
pprB-up-HindIII-F	GAGATAAGCTTTTCCGAGCATGAGCTGACATCCC	
pprB-up-BamHI-R	GAGATGGATCCGATCAAGACGCTGAAATGCCG	
pprB-dn-BamHI-F	GAGATGGATCCCACTGACAGGCGCGATGG	
pprB-dn-XbaI-R	GAGATTCTAGACGGATGGAATGGGCTTGATC	
pprA-upF	GGGGATGTGCTGCAAGGCGATTAGGTCATACGCTCCATTTGC	
pprA-upR	GAACCACGAACAAGCGGTCCTGTTCGTCCATC	
pprA-dnF	CGAACAGGACCGCTTGTTCGTGGTTCGCTTGCCG	
pprA-dnR	GATCCTCTAGAGTCGACCTGCAGCGATGGTCTTCTCCCTGCTC	
bapA-upF	GGGGATGTGCTGCAAGGCGATTATGTCCACGCTCAATAGTCGC	
bapA-upR	CCGAATCGCCCTTAGCCGCAGAAGAAATACT	
bapA-dnF	TCTTCTGCGGCTAAGGGCGATTCGGGTAGCGT	
bapA-dnR	GATCCTCTAGAGTCGACCTGCAGCGGCAGAGCAGCAACAAGC	
cupE-upF	GGGGATGTGCTGCAAGGCGATTACGGCTTCGTCTACACCTTCA	
cupE-upR	GTGTTGCTGGTGGTGGTGCTGCACTGGATCTGGATAT	
cupE-dnF	TCCAGTGCAGCACCACCACCAGCAACACCCAGG	
cupE-dnR	GATCCTCTAGAGTCGACCTGCAGTTCCCGCATTCAGCAGTTCTAT	
relA-upF	CACACAGGAAACAGCTATGACATGATCGAGCCGGAAGAATGGG	
relA-upR	TGATACTGCCGTCGGTGTTGA	
relA-dnF	GTCAACACCGACGGCAGTATCATGAGGCGAGGCGGAAACA	
relA-dnR	GACGTTGTAAAACGACGGCCAGTCGTCGGCAGCATCAACCAGGC	
Transcriptional reporter plasmid cloning primers		
PpprB-F	AGAGGGAGGGCAAGTCCAACCAGTTAACTGGCTTATCCTGGGC	
PpprB-R	CTCTATAGTGAGTCGGGATCGCTAGTGGTTACGCAACGGTAGC	
PrcpC-F	AGAGGGAGGGCAAGTCCAACCAGTTTACGGCAATCAGAGCCAC	
PrcpC-R	CTCTATAGTGAGTCGGGATCGCTAGGGCCGATGGATACGCCGAG	
Pflp-F	AGAGGGAGGGCAAGTCCAACCAGTTTCACGCACGAAGAGCATC	
Pflp-R	CTCTATAGTGAGTCGGGATCGCTAGTACGGCAATCAGAGCCAC	
PcupE-F	AGAGGGAGGGCAAGTCCAACCAGTTATCCTCTGCCTGCTGTTC	
PcupE-R	CTCTATAGTGAGTCGGGATCGCTAGACGCTGCCGTTGATGATG	
PpprB-mut2-F	TGCTTATGGCCGGTACCATTCGGTATGGCTGC	
PpprB-mut2-R	CGAATGGTACCGGCCATAAGCATATAGTCGCTAAGTG	
PpprB-mut1-F	GCGGAAAACCTCACTTAGCGAGGGTATGCTTATGGCTAATACCATTCGG	
PpprB-mut1-R	TCGCTAAGTGAGGTTTTCCGC	
PUCP20-F	CTGTCGTGCCAGCTGCATT	
PUCP20-R	AATGCAGCTGGCACGACAG	

### Carbon deprivation experiment of transcriptional reporter strains.

Overnight cultures of P. aeruginosa strains in FABS supplemented with 30 mg/ml gentamicin (FABSgen) were diluted 100× and grown to the exponential phase (OD_600_ of ∼0.6) in 1 ml FABSgen medium. For each sample, 900 μl of bacterial culture (OD_600_ of ∼0.6) was used for CSS treatment. Cells were washed once with 1 ml FAB and resuspended in 1 ml FAB plus 30 μg/ml gentamicin (FABgen) in a 5.5-ml polystyrene tube (Falcon 352054); the suspensions were cultivated for 5 h with shaking (250 rpm). Gentamycin addition does not change the CSS-induced transcriptional response of PprB-regulated genes (see Fig. S7 in the supplemental material). For carbon deprivation in PApprB1 and PApprB2 strains, overnight cultures were diluted 100× in FABSgen plus 0.4% (wt/vol) l-arabinose and grown to the exponential phase before following the same procedures noted above.

### Monitoring *pprB* or *rpoS* expression under other stress conditions.

Overnight cultures of *pprB* or *rpoS* transcriptional reporter strains in FABS supplemented with 30 μg/ml gentamicin (FABSgen) were diluted 100× and grown to the exponential phase (OD_600_ of ∼0.6) in 1 ml FABSgen medium. Cells were washed once with pure FAB and then diluted 10× into 1 ml acetate medium (FABSgen plus 200 mM acetate), –N medium (FABSgen medium without ammonium sulfate), –P medium (FABSgen medium with 10% KH_2_PO_4_ and 10% Na_2_HPO_4_ added to the original medium), +3M NaCl medium (FABSgen plus 3 M NaCl), or +10% ethanol medium (FABSgen plus 10% [vol/vol] ethanol) and cultivated for 5 h with shaking (250 rpm). SfGFP fluorescence of cells was then measured by microscopy as mentioned below.

### Construction of gene deletion or complementary mutants in P. aeruginosa.

PCR was used to generate 1,000-bp DNA fragments upstream (Up) or downstream (Dn) from the *pprA*, *pprB*, *flp*, *cupE*, *bap*, and *relA* genes. The primer pairs are listed in Table 1. The Up and Dn DNA fragments for *pprA*, *cupE*, *bap*, and *relA* were ligated together using overlap extension PCR and then inserted into the pex18gm vector via Gibson assembly. The recombinant plasmids were introduced into P. aeruginosa through electroporation, and the deletion mutants were obtained by double selection on LB agar supplemented with gentamicin (30 μg/ml) and NaCl-free LB agar containing 15% sucrose at 37°C (49). The Up and Dn DNA fragments for *cbrAB*, *pprB*, and *flp* were digested and cloned into pex18ap at HindIII-XbaI sites together with *aacC1*. The recombinant plasmids were electroporated into P. aeruginosa, and deletion mutants were obtained by selection on LB agar supplemented with gentamicin (30 μg/ml) containing 5% sucrose at 37°C. Then, the pFLP2 system was used to delete the *aacC1* cassette (50). The miniTn*7* system (51) was used to construct the complementary *pprB* and *rpoS* mutants in P. aeruginosa. PCR fragments of *pprB* coding sequences and *araC-P_BAD_* were inserted into the miniTn*7* vector via Gibson assembly, generating *P_BAD_-pprB*-Tn*7*. A mutated *pprB* (*pprB* D60A) complementary plasmid was cloned from *P_BAD_-pprB*-Tn*7* by primer pair pprB-D60A-F/pprB-D60A-R, generating *P_BAD_*-pprB(D60A)-Tn*7*. PCR fragments of the *rpoS* coding sequence, together with the *rpoS* promoter sequence, were inserted into the miniTn*7* vector via Gibson assembly, generating *P_rpoS_-rpoS*-Tn*7*. These resultant plasmids were introduced to the *pprB* or *rpoS* mutant strains through electroporation, and the transconjugants were selected on 1.5% LB agar plates supplemented with 30 μg/ml gentamicin. The gentamicin resistance cassette in the complementary strains was then deleted according to a standard protocol (50).

### Construction of transcriptional reporters in P. aeruginosa.

The SfGFP fusion plasmid used to measure the promoter activity of multigenes is a derivative of the vector pUCP20, here named pUCPgfp. *sfgfp*, *cyofp*, and terminator fragments were amplified using PCR and inserted together into pUCP20 via Gibson assembly, generating pUCPgfp (Fig. S1). The resultant genetic organization was RNAseIII-RBS2-*sfgfp*-T_0_T_1_-J23102-RBS2-*cyofp*-T-pUCP20, where J23102 is a constitutive promoter (http://parts.igem.org/Promoters/Catalog/Anderson). Constitutively expressed CyOFP provides a standard cell identification protocol to avoid the influence of fluorescence halo effect in different transcriptional reporters and also to avoid failures in cell mask determination when SfGFP intensities are weak. Cell masks identified from CyOFP images overlap well with cells in SfGFP images, thereby enabling the quantification of transcriptional activity for target promoters (see Fig. S8). To construct the transcriptional fusion plasmids, promoter regions of *pprB*, *flp*, *rcpC*, and *cupE* were amplified by PCR from the PAO1 genomic DNA. The primer sets are noted in Table 1. Next, each fragment was cloned into pUCPgfp right before the RNAseIII site via Gibson assembly. The reporter plasmids were introduced into P. aeruginosa through chemical transformation. To introduce site mutations in the *pprB* promoter region of *pprB* transcriptional reporters, the wild-type *pprB* reporter plasmid was used as a template. Two fragments were amplified: the primer pairs PpprB-mut1-F/PUCP20-R and PpprB-mut1-R/PUCP20-F were used for RpoS binding site mutation, and PpprB-mut2-F/PUCP20-R and PpprB-mut2-R/PUCP20-F were used for PprB binding site mutation. Then, the fragments were ligated together via Gibson assembly, generating p*pprB*-mutRpoS-pUCPgfp (p*pprB* mut1-*sfgfp*) and p*pprB*-mutPprB-pUCPgfp (p*pprB* mut2-*sfgfp*) plasmids. These two plasmids were introduced into P. aeruginosa strains through electroporation. All transconjugants were selected on 1.5% LB agar plates supplemented with 30 μg/ml gentamicin.

### Imaging of single cells of different promoter reporter strains and data analysis.

The bacterial culture samples were pipetted and loaded on a 2% (wt/vol) agarose FAB pad. Then, the pad was flipped onto a 0.15-mm cover glass so that the bacteria were sandwiched and lay flat between the agarose pad and the cover glass. Fluorescence images were acquired with a confocal microscope (IX-81; Olympus) equipped with a 100× oil lens objective and an electron multiplying charge-coupled-device (EMCCD) camera (Andor iXon897). Twenty-five image fields of each sample were snapped, from which more than 500 cells were imaged. In each image field, two images were acquired, one SfGFP image and one CyOFP image. SfGFP and CyOFP were both excited using a 488 nm laser, and the fluorescence was collected through two emission filters, sized at 524 ± 25 nm and 607 ± 25 nm. Data analysis was conducted using an image processing algorithm coded using MATLAB. Cell masks were obtained from the CyOFP images, and then the SfGFP fluorescence of cells was measured by counting the mean intensities within corresponding cell masks in the SfGFP images.

### RNA-seq experiment.

Six parallel samples (50 ml each) were prepared, in which the overnight culture of PAO1 was diluted 50× in FABS medium and grown until the exponential phase (OD_600_ of ∼0.6) at 37°C under shaking conditions. Three samples were stored at −80°C, while the remaining three samples were washed 3 times with FAB and finally resuspended in 50 ml FAB. These suspension cultures were cultivated for a further 6 h with shaking at 37°C and stored at −80°C. Total RNA was extracted by using TRIzol reagent (catalog number 15596018; Invitrogen) and zirconia beads, precipitated with isopropanol at −20°C for 1 h, washed with 70% ethanol, and dissolved in diethyl pyrocarbonate (DEPC)-treated water (AM9915; Ambion), according to the manufacturer’s protocol. The concentration, RNA integrity number (RIN), 23S/16S, and size of total RNA were detected by using Agilent 2100 Bioanalyzer (Agilent RNA 6000 Nano kit). A NanoDrop was used to determine the purity of samples. mRNA was isolated by using an NEBNext Poly(A) mRNA magnetic isolation module according to the manufacturer’s protocol. The total RNA samples were then used for library construction and sequencing. Libraries were sequenced on an Illumina HiSeq 2000 machine. The 100-nucleotide-long reads were mapped using HISAT software to the P. aeruginosa PAO1 RefSeq genome (NC_002516.2). The functional annotation information of P. aeruginosa was obtained from the Pseudomonas Genome Database (https://www.pseudomonas.com). Genes with more than 5-fold transcriptional change under CSS are shown in Table S1.

### Aggregation assay.

Overnight cultures of the *pprB* overexpression (in pJN105) or wild-type strains were diluted 100× in FABSgen medium supplemented with 0.02% (wt/vol) l-arabinose and grown to the exponential phase (OD_600_ of ∼0.6) at 37°C. To measure the size of bacterial aggregates, 200 μl of each bacterial suspension was transferred to a 4-channel dish (D35C4-20-1-N; Cellvis) and left to stand for 10 min at room temperature. The bacterial aggregates were monitored under a bright-field microscope equipped with a 60× oil lens objective. One hundred images containing at least 200 bacterial aggregates were obtained every time. Three parallel experiments were conducted for each sample. The sizes of aggregates were recorded using ImageJ software. For proteinase treatment, 20 μl proteinase K (R7012; Tiangen) was added to 1 ml bacterial culture at exponential phase and incubated for 30 min at 37°C.

### Microfluidic experiment.

For the microfluidic experiment of the *pprB* overexpression (in pJN105) strains, the culture conditions were the same as those for the aggregation assay. For the microfluidic experiments of the PAO1 and *pprB* mutant strains, the culture condition was the same as those for the carbon starvation experiment for transcriptional reporter strains, without the addition of gentamicin. The microchip platform was fabricated with polydimethylsiloxane (PDMS) (Sylgard 184; Dow Corning) using standard soft lithography methods (52). Wafers were coated with SU-8 photoresist (MicroChem Inc., Newton, MA, USA) to form film depositions of up to 20 μm. The mold contained three parallel microchannels (length, 3 cm; width, 300 μm; height, 20 μm) and was firmly stuck to a heat-tolerant plastic tray. Ten milliliters of the PDMS mixture, consisting of cross-linker and prepolymer PDMS (1:10 [wt/wt]), was added to the tray and baked at 80°C for 2 h. The structure was then treated with a plasma cleaner (3 min) and bonded to a glass slide (Thermo Fisher Scientific Inc.; length, 55 mm; width, 24 mm; thickness, 0.17 mm). In total, 0.5 ml of bacterial culture was injected into the channel for each experiment. The FAB medium was in a 10-ml gas-tight syringe, and fluid flow was driven by a syringe pump (Phd2000; Harvard Apparatus, Holliston, MA).

### CFU measurement.

P. aeruginosa cultures cultivated under CSS for 0, 6, 12, and 36 h were diluted up to 5,000-fold with FAB medium and plated in triplicates onto LB agar plates. Colonies were counted after a 24-h incubation at 37°C.

### Colony biofilm experiment.

*pprB*, *rcpC*, *flp*, and *cupE* reporter strains of wild-type P. aeruginosa were grown to the exponential phase in FAB medium containing 30 mM succinate and 30 μg/ml gentamicin, and then 2 μl bacterial culture was gently dropped onto 1.5% agar plates containing the same medium. After the liquid on the culture plate evaporated, the plates were incubated upside down at 37°C for 6, 12, 24, 48, and 72 h. Cells were scratched from the surfaces of biofilm colonies and resuspended in FAB medium before undergoing fluorescence measurement via microscopy.

### Data availability.

All sequencing results were deposited in NCBI SRA under BioProject number PRJNA550173 and BioSample accession numbers SAMN12109780 to SAMN12109785.

## Supplementary Material

Supplemental file 1

Supplemental file 2
