# Supplementary material for: Carbon Starvation Induces the Expression of PprB-Regulated Genes in Pseudomonas aeruginosa
Source: Appl Environ Microbiol. 2019 Oct 30;85(22):e01705-19. doi: 10.1128/AEM.01705-19 (PMC6821963; doi:10.1128/AEM.01705-19)
Supplement: Supplemental file 1 [file AEM.01705-19-s0001.pdf]

Supplemental Text

Carbon starvation induces the expression of PprB-regulated genes in *Pseudomonas aeruginosa*

Congcong Wang,[a] Wenhui Chen,[b] Aiguo Xia,[b,c] Rongrong Zhang,[b,c] Yajia Huang,[c] Shuai Yang,[b,c] Lei Ni,[b,c,#] and Fan Jin,[b,c,#]

a. Department of Chemical Physics, University of Science and Technology of China, No. 96, JinZhai Road Baohe District, Hefei, Anhui 230026, P. R. China
b. Hefei National Laboratory for Physical Sciences at the Microscale, University of Science and Technology of China, No. 96, JinZhai Road Baohe District, Hefei, Anhui 230026, P. R. China
c. CAS Key Laboratory of Quantitative Engineering Biology, Shenzhen Institute of Synthetic Biology, Shenzhen Institutes of Advanced Technology, Chinese Academy of Sciences, Shenzhen 518055, China

Congcong Wang and Wenhui Chen contributed equally to this article.

#, Address correspondence to Lei Ni, nilei@mail.ustc.edu.cn, or Fan Jin, fjinustc@ustc.edu.cn.

Growth curve measurement. Overnight cultures of PAO1-pJN105 cells in FABSgen or pprBpJN-PAO1 cells in FABSgen + 0.02% L-arabinose were 100× diluted into the same media and were grown for 10 hours with shaking at 37 ℃. OD_{600} values were recorded every hour. Three parallel experiments were performed for each strain. Due to the enhanced cell-cell adhesion of PprB expressed cells, overnight culture of this strain contains lots of cell clusters at the bottom of the culture tube, this results in a relatively fewer cells in the supernatant compared to that in wild type. Thus, when we took a certain volume (10 μL) of overnight culture for extended culture and growth curve measurement, the initial cell number of PprB overexpressed strain is less. As the resolution of our absorption spectrophotometer is poor at OD600<0.05, the resulting growth curve of PprB overexpression strain displayed a lag phase in the first three hours (Fig. S4).

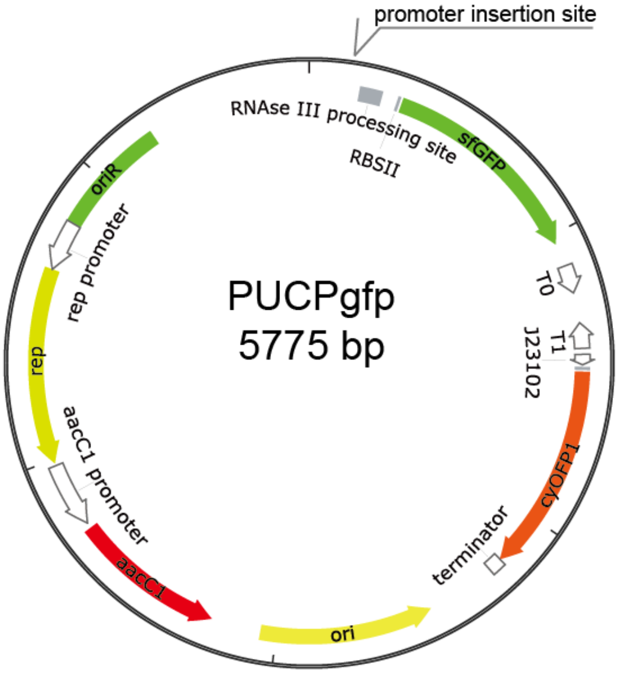

Figure S1: Functional map of plasmid PUCPgfp. PUCPgfp is derived from PUCP20, the promoter sequences were inserted before the RNAse III processing site.

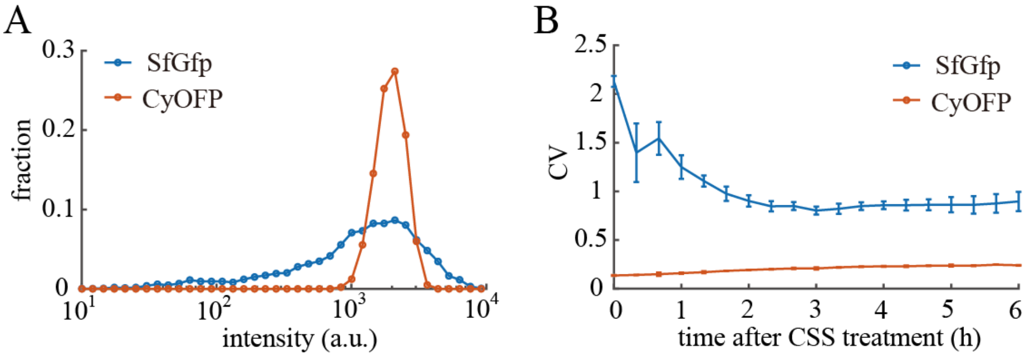

Figure S2: Heterogeneity of flp transcription among cells. (A), Distribution of SfGFP (expressed by *flp* promoter) and CyOFP (expressed by J23102 promoter) fluorescence intensity among cells after 4-hour CSS treatment. B, Coefficient of variations (CV) of the distribution of SfGFP and CyOFP fluorescence intensity after CSS treatment, data are from three independent experiments and shown as the

mean ± s.d.

flp

-83 TGCAAAACGCCATCATCGGCCTAGCCTCAGCGTGGAGGCAGGGCTC

cupE

-198 CAGAGGCTTATTCGCGCTGCTATATAGCCAGTCAAGCATGACTGGC

rcpC

-280 GGCGGGTTACAAGGCTTGGACTACATCTAATGACAAATTGACATTGAT

Figure S3: Promoter regions of the *cupE, rcpC and flp* genes. Putative RpoS binding sites are framed.

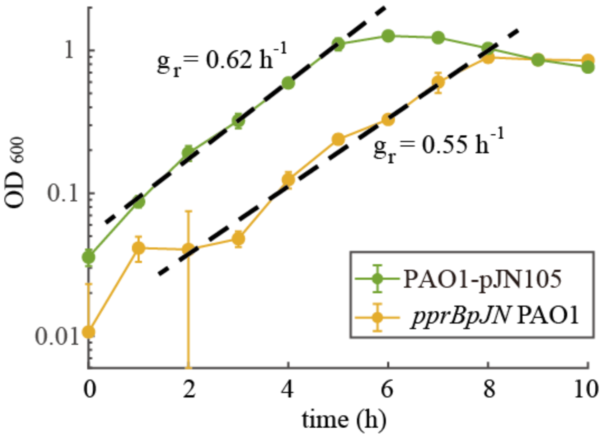

Figure S4: Growth curves of wild type and the PprB overexpression strains. Data are from three independent experiments and shown as the mean ± s.d. Growth rates (g_r) are fitted from data at exponential phase using linear fit method, the resulting g_r are indicated in the graph.

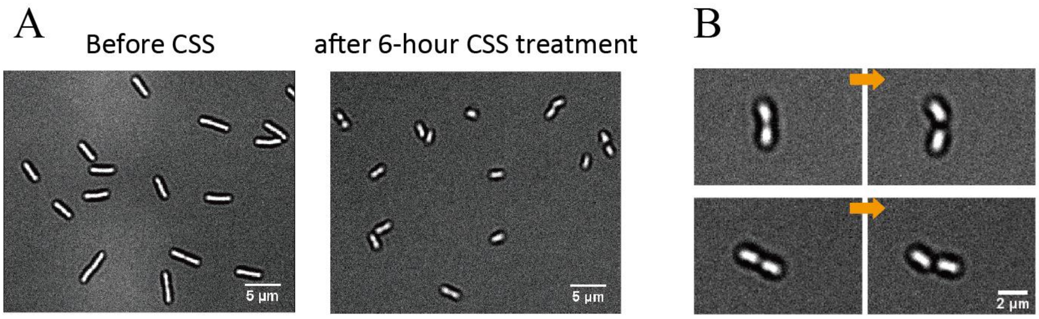

Fig. S5: Bacteria can still divide after carbon deprivation. A, Bright-field microscopy image of *pprB* mutant cells before and after 6 hours of CSS treatment. B, Two division events of *pprB* mutant cells under CSS.

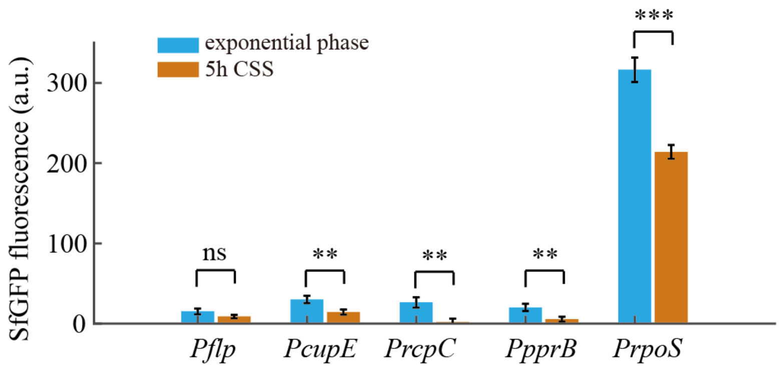

Figure S6: Expression values of *flp, cupE, rcpC, pprB, and rpoS* transcriptional reporters in *relA* mutant strain under exponential phase or after 5 hours of carbon deprivation. Statistical analysis used pairwise comparisons (t-test) of data in control experiment to data in stress conditions. ***P < 0.001, **P < 0.01, ns P>0.05.

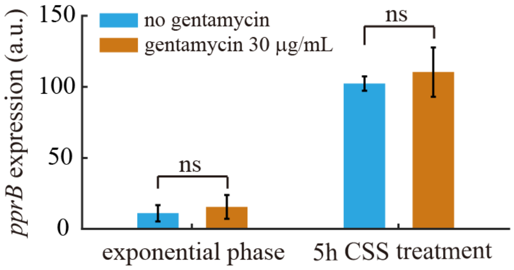

Fig. S7: Gentamycin addition does not affect the induction of PprB-regulated gene expression under CSS. Expression values of *pprB* transcriptional reporter with or without gentamycin addition at exponential phase or after 5 hours of CSS treatment.

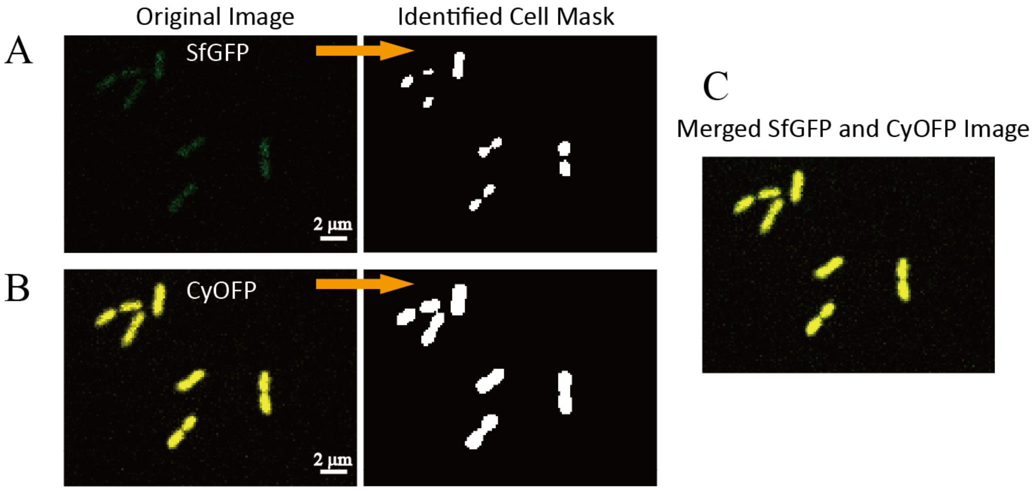

Fig. S8: Bacterial cell identification from fluorescent images. (A) Cell mask determination using SfGFP image of PpprB reporter under carbon abundant condition. (B) Cell mask determination using CyOFP image of PpprB reporter. (C) Merged SfGFP and CyOFP image of PpprB reporter.
